# EORTC Group Phase II Study of Oral Etoposide for Pretreated Soft Tissue Sarcoma

**DOI:** 10.1080/13577149778371

**Published:** 1997-06

**Authors:** H. Jan Keizer, Derek Crowther, Ole Steen Nielsen, Allan T. Van Oosterom, Javier Hornedo Muguiro, Christine Van Pottelberghe, Renier Somers, Thomas Tursz

**Affiliations:** 1 Leiden University Hospital The Netherlands; 2 University of Manchester UK; 3 Aarhus University Hospital Denmark; 4 University Hospital of Antwerp Belgium; 5 Madrid Hospital Universitario Spain; 6 EORTC Data Center Belgium; 7 Netherlands Cancer Institute The Netherlands; 8 Institut Gustave-Roussy France; 9 Leiden University Medical Center Department of Clinical Oncology PO Box 9600 (K1-P) RC Leiden NL-2300 The Netherlands

## Abstract

*Purpose*. This study investigates the efficacy and toxicity of daily oral etoposide in chemotherapy for non-heavily pretreated advanced and metastatic soft tissue sarcoma (STS).

*Subjects*. Twenty-seven patients with progressive and measurable disease were treated. Median age was 53 years (range 20–71 years) and performance status WHO 0 or 1. Histologies included mainly leiomyosarcoma (8), malignant fibrous histiocytoma (4), rhabdomyosarcoma (4), liposarcoma (2) and synovial sarcoma (2). Fifteen patients had received prior radiotherapy, of whom three included sites with haematopoiesis. All patients had received prior chemotherapy, including adjuvant therapy (7) and mostly consisted of one two-drug schedule (ifosfamide and doxorubicin) or two single-drug regimens.

*Methods*. Chemotherapy consisted of etoposide (VP16-213), 50 mg m^-2^ day^-1^ × 21 q 4 weeks. Blood cell counts were done weekly. Dose reductions and a maximum delay of 2 weeks was allowed depending on cell counts during treatment and at the start of a new 4-week treatment cycle.

*Results*. No objective response was observed. Progressive disease was observed after two treatment cycles in 17/27 patients (68%) and after three cycles in 22/27 patients (81%). The other patients received three to five cycles. Twenty-four patients went off study due to progressive disease. Grade 3 and 4 neutropenia was observed in eight and one patients, respectively. Thrombocytopenia grade 3 was seen in two patients. Non-haematological toxicity grade 3 (nausea, diarrhoea or alopecia) was observed in three patients, and grade 4 (dyspnea, hypotension or haemorrhage) in three patients.

*Discussion*. No objective response was obtained. Oral etoposide at a dose of 50 mg m^-2^ day^-1^ × 21 q 4 weeks is inactive in chemotherapy of pretreated STS. Disease progression occurred within three cycles in the majority (81%) of patients. Toxicity of this regimen in non-heavily pretreated patients is low.


*Sarcoma* (1997) 1, 99–101

ORIGINAL ARTICLE

### EORTC Group phase II study of oral etoposide for pretreated soft tissue sarcoma

H. JAN KEIZER,[1] DEREK CROWTHER,[2] OLE STEEN NIELSEN,[3] ALLAN T. VAN OOSTEROM,[4] JAVIER HORNEDO MUGUIRO,[5] CHRISTINE VAN POTTELBERGHE,[6] RENIER SOMERS[7] & THOMAS TURSZ[8]

[1]*Leiden University Hospital, The Netherlands,* [2]*University of Manchester, UK,* [3]*Aarhus University Hospital, Denmark,* [4]*University Hospital of Antwerp, Belgium,* [5]*Madrid Hospital Universitario, Spain,* [6]*EORTC Data Center, Belgium,* [7]*Netherlands Cancer Institute, The Netherlands &* [8]*Institut Gustave-Roussy, France*

**Abstract**

*Purpose.* This study investigates the efficacy and toxicity of daily oral etoposide in chemotherapy for non-heavily pretreated advanced and metastatic soft tissue sarcoma (STS).

*Subjects.* Twenty-seven patients with progressive and measurable disease were treated. Median age was 53 years (range 20–71 years) and performance status WHO 0 or 1. Histologies included mainly leiomyosarcoma (8), malignant fibrous histiocytoma (4), rhabdomyosarcoma (4), liposarcoma (2) and synovial sarcoma (2). Fifteen patients had received prior radiotherapy, of whom three included sites with haematopoiesis. All patients had received prior chemotherapy, including adjuvant therapy (7) and mostly consisted of one two-drug schedule (ifosfamide and doxorubicin) or two single-drug regimens.

*Methods.* Chemotherapy consisted of etoposide (VP16-213), 50 mg $m^{-2}$ $day^{-1}$ × 21 q 4 weeks. Blood cell counts were done weekly. Dose reductions and a maximum delay of 2 weeks was allowed depending on cell counts during treatment and at the start of a new 4-week treatment cycle.

*Results.* No objective response was observed. Progressive disease was observed after two treatment cycles in 17/27 patients (68%) and after three cycles in 22/27 patients (81%). The other patients received three to five cycles. Twenty-four patients went off study due to progressive disease. Grade 3 and 4 neutropenia was observed in eight and one patients, respectively. Thrombocytopenia grade 3 was seen in two patients. Non-haematological toxicity grade 3 (nausea, diarrhoea or alopecia) was observed in three patients, and grade 4 (dyspnea, hypotension or haemorrhage) in three patients.

*Discussion.* No objective response was obtained. Oral etoposide at a dose of 50 mg $m^{-2}$ $day^{-1}$ × 21 q 4 weeks is inactive in chemotherapy of pretreated STS. Disease progression occurred within three cycles in the majority (81%) of patients. Toxicity of this regimen in non-heavily pretreated patients is low.

**Key words:** *soft tissue sarcoma, pretreated, chemotherapy, etoposide.*

### Introduction

Etoposide (VP16/213) is a major cytostatic agent with a broad antitumour spectrum.[1] In clinical practice, its schedule dependency has been established.[1,2] Frequently used schedules are daily administrations for 3 to 5 days or at days 1, 3 and 5. This schedule dependency prompted clinicians to examine prolonged administration schedules, in particular daily oral dosing, for 14–21 days in 3- or 4-week cycles. This chronic administration proved to be active in several malignancies including solid tumours such as breast cancer, gastric cancer, mesothelioma and Kaposi sarcoma.[1]

Interestingly, some tumours, resistant to standard treatment schedules, responded again following chronic oral schedules. The EORTC Soft Tissue and Bone Sarcoma Group and others found no significant activity of VP16 intravenously at dosages of 120–130 mg $m^{-2}$, days 1–3 or days 1–5, respectively, every 3 weeks.[3,4] However, most of these patients were heavily pretreated. The Scandinavian Sarcoma Group used a combination of ifosfamide (450 mg $m^{-2}$) and etoposide (600 mg $m^{-2}$) as a continuous infusion. The 42% response rate was considered promising by the authors, who also claimed the effectivity of continuous etoposide administration.[5]

Chronic administration of oral etoposide, planned dose 150 mg/day for 15 days q 3 weeks, was studied in 15 heavily pretreated advanced soft tissue sarco-

Correspondence to: H. J. Keizer, Leiden University Medical Center, Department of Clinical Oncology, PO Box 9600 (K1-P), NL-2300 RC Leiden, The Netherlands. Tel: +31 71 5263486; Fax: +31 71 5266760.

1357-714X/97/020099-03 $9.00 ©1997 Carfax Publishing Ltd mas (STS).[6] No objective responses were observed. Half of the patients received one cycle only, while the others received two cycles. Frequently, the daily dose or the number of treatment days had to be limited. Based on data of objective responses after prolonged administration of oral etoposide in non-heavily pretreated patients, reported by some members, the EORTC-STS Group initiated a phase II study in pre-treated patients who had received one two-drug schedule or two single drugs only.

### Subjects and methods

### *Subjects*

Twenty-seven patients with histologically proven, progressive, measurable and advanced or metastatic STS were entered: 15 females, 12 males; median age 53 years (range 20–71 years); WHO performance status grade 0 (10) or grade 1 (17). Most frequent histologies were leiomyosarcoma (8), malignant fibrous histiocytoma (4), rhabdomyosarcoma (4), liposarcoma (2) and synovial sarcoma (2). Fifteen patients had received prior radiotherapy, of whom three included sites with haematopoiesis.

All patients had received prior chemotherapy, including adjuvant therapy (7). This chemotherapy consisted of one two-drug schedule (usually ifosfamide and doxorubicin) or two single-drug regimens. Further in- and exclusion criteria were similar to the standard phase II criteria of the EORTC-STS Group.

### *Treatment*

Chemotherapy consisted of etoposide (VP16-213) 50 mg $m^{-2}$ $day^{-1}$ orally for 21 consecutive days, repeated every 4 weeks. Weekly haematological parameters were determined. If at day 1 of a treatment cycle, white blood cells (WBC) or platelets were below $3 \times 10^9$/l or $100 \times 10^9$/l, respectively, treatment was delayed until recovery for a maximum of 2 weeks. If no recovery took place, a dose reduction was made.

Etoposide treatment was discontinued for 1 week if during treatment the 3-week cycle WBC fell below $2 \times 10^9$/l or platelets below $75 \times 10^9$/l, until recovery (maximum delay 2 weeks). Dose reductions at the start of a treatment course were as follows: WBC 2.5–2.9 $\times 10^9$/l or platelets 75–99, 25% dose reduction; WBC 2.0–2.4 $\times 10^9$/l or platelets 50–74, 50% dose reduction; WBC $< 2.0 \times 10^9$/l or platelets $< 50$, off study.

### Results

No objective response was obtained. After two cycles of treatment, progression was already present in 17/27 patients (68%) and after three cycles in 22/27 patients (81%). The five remaining patients had ‘no change’ as their best response and received three to five cycles of treatment. A total of 24 patients went off study due to tumour progression.

Only one patient experienced grade 4 neutropenia (patient went off study), eight patients had grade 3 neutropenia, without any fever. Grade 3 thrombocytopenia was observed in two patients. One patient went off study after 2 weeks with a platelet count of $54 \times 10^9$/l. Grade 4 non-myelotoxicity occurred in three patients (one pulmonary embolism, one acute respiratory failure after the first cycle and one gastrointestinal bleeding, without thrombocytopenia—this patient went off study). These latter toxicities were definitely considered to be unrelated to the chemotherapy.

### Discussion

The data presented show that in 27 patients who had been previously treated with two single-drugs regimens or one two-drug combination, a regimen of 3 weeks oral etoposide (50 mg $m^{-2}$ $day^{-1} \times 21$, q 4 weeks) failed to achieve an objective response. Moreover, 22/27 patients (81%) already showed progressive disease after two or three courses of treatment. The toxicity of this regimen is low, which may be explained by the relatively low dose and the previously received chemotherapy.

Yet, with a similar regimen, Hainsworth *et al.*[7] in refractory or resistant patients, reported 2/3 partial responses. Using higher dosages (3-weekly intravenous 130 mg $m^{-2}$) the EORTC-STS Group reported 1/26 patients with a partial response.[3] The use of higher dosages in a daily oral schedule was reported by Kampe *et al.*[6] and resulted in severe mucositis, gastrointestinal side-effects and myelosuppression. No response was seen in the 15 heavily pretreated patients (no further specifications were given). Discontinuation of treatment and dose reductions were frequently needed in this latter study.

There is hardly any data on etoposide as a single agent, in first-line treatment for STS. The Scandinavian Sarcoma Group used a combination of etoposide and ifosfamide (both as a continuous infusion) and found a 42% objective response rate. In second line studies the majority of reports, including our own, indicate that this drug does not have significant efficacy. The effect of etoposide as a first-line single agent (oral or intravenous) in STS has yet to be determined.

### References

1 Hainsworth JD, Greco FA. Etoposide: twenty years later. *Ann Oncol* 1995; 6:325–41.
2 Slevin ML, Clark PI, Osborne RJ, *et al.* A randomized trial to evaluate the effect of schedule on the activity of etoposide in small cell lung cancer. *J Clin Oncol* 1990; 7:1333–40.

3 Dombernowsky P, Buesa J, Pinedo HM, *et al.* VP-16 in advanced soft tissue sarcoma: a phase II study of the EORTC Soft Tissue and Bone Sarcoma Group. *Eur J Cancer Clin Oncol* 1987; 23:579–80.
4 Welt S, Magill GB, Sordillo PP, *et al.* Phase II trial of VP-16-213 in adults with advanced soft tissue sarcomas. *Proc Am Soc Clin Oncol* 1983; 3:234.
5 Sæter G, Talle K, Solheim ØP. Treatment of advanced high-grade soft tissue sarcoma with ifosfamide and continuous infusion etoposide. *Cancer Chemother Pharmacol* 1995; 36:172–5.
6 Kampe CE, Löwenbraun S, Foster J, *et al.* Oral etoposide in treatment of advanced refractory sarcomas. *J Natl Cancer Inst* 1992; 84:1836–7.
7 Hainsworth JD, Johnson DH, Frazier SR, *et al.* Chronic daily administration of oral etoposide—a phase I trial. *J Clin Oncol* 1989; 8:396–401.
